# Processing Impact on In Vitro and In Vivo Performance of Solid Dispersions—A Comparison between Hot-Melt Extrusion and Spray Drying

**DOI:** 10.3390/pharmaceutics13081307

**Published:** 2021-08-21

**Authors:** Yongjun Li, Amanda K. P. Mann, Dan Zhang, Zhen Yang

**Affiliations:** Department of Pharmaceutical Sciences, MRL, Merck & Co., Inc., Kenilworth, NJ 07033, USA; zhen.yang@hansohbio.com

**Keywords:** amorphous solid dispersion, hot-melt extrusion, spray drying, dissolution, PK comparison

## Abstract

Presently, a large number of drug molecules in development are BCS class II or IV compounds with poor aqueous solubility. Various novel solubilization techniques have been used to enhance drug solubility. Among them, amorphous solid dispersions (ASD), which convert a crystalline drug into an amorphous mixture of drug and polymer, have been demonstrated to be an effective tool in enhancing drug solubility and bioavailability. There are multiple ways to produce amorphous solid dispersions. The goal of the present study is to investigate two commonly used processing methods, hot-melt extrusion (HME) and spray drying, and their impact on drug bioperformance. The amorphous solid dispersions of a model compound, posaconazole (25% drug loading) in HPMCAS-MF, were successfully manufactured via the two processing routes, and the physicochemical properties, in vitro and in vivo performance of the resulting ASDs were characterized and compared. It was found that in vitro drug release of the ASDs from two-stage dissolution was significantly different. However, the two ASDs showed similar in vivo performance based on cynomolgus monkey PK studies. A mechanistic understanding of the in vitro and in vivo behaviors of the solid dispersions was discussed.

## 1. Introduction

Poorly soluble drugs continue to pose challenges to drug development, including the inability to achieve target exposures in toxicology studies, low and variable PK in clinical trials, food effect, and pill burden to patients [1]. Various novel solubilization techniques have been developed to overcome these challenges [1,2]. Among these techniques, amorphous solid dispersion (ASD) has emerged as an effective approach to increasing the bioavailability of low solubility compounds [3,4,5,6]. Due to its versatility in applying to a wide range of compounds, ASDs have been increasingly used to support drug development and have successfully delivered several commercial products [7,8,9]. Many studies were conducted to understand the mechanism of bioavailability improvement through solid dispersions. Several factors contribute to the increased bioavailability of ASDs [10,11,12,13,14,15]. First, the conversion of the crystalline drug into its amorphous form results in a higher apparent solubility, and subsequently better bioperformance, especially when absorption of the drug is fast [10,11]. Second, the dispersion of API in the polymer matrix impacts drug dissolution through the improvement of API wettability, particle size reduction, and stabilization of the dissolved drug [12,13,14,15]. The ability of an amorphous dispersion to accelerate the dissolution rate and maintain the concentration of the drug in solution is often referred to as the “spring” and “parachute” mechanism and is expected to result in favorable bioperformance [16]. The “spring” is ascribed as a drug in a higher energy state, such as the amorphous phase, which quickly releases into the solution and reaches supersaturation. In this metastable state, however, the drug could crash out of the solution and precipitate as a crystalline solid, resulting in a rapid decrease in drug concentration with limited benefit for bioavailability. To realize the full advantage of ASDs, efforts were directed to inhibit or retard the crystallization and precipitation process to extend the supersaturation or slow down the decrease in drug concentration, referred to as the “parachute” effect. Therefore, the rational design of ASD composition was largely based on the functional roles of the excipients that could facilitate the fast release of drug as well as extend the supersaturation [17,18].

The composition of the ASD is expected to impact the properties, stability, and dissolution of the material. However, the processing route to generate the material can also play a key role [19,20,21,22]. A number of routes have been used to generate ASDs, including spray drying, hot-melt extrusion (HME), and co-precipitation [23,24,25,26,27]. Spray drying is a process where the drug and polymer are dissolved in a solvent, and the solvent is rapidly evaporated as the solution is atomized into the droplets that move through a drying chamber. The particles from spray-dried dispersions (SDD) are typically spherical and in the micron size range [28]. For efficient spray drying, the high solubility of the drug and polymer in a common solvent that has a relatively low boiling point is required [29,30]. Typically, SDDs are easy to make at a small scale, which is especially important and beneficial at the early stages of development [31]. In addition, this process is amenable to a wide variety of polymers [31]. However, the physicochemical properties of the SDDs may be inconsistent when the process is scaled up, and the SDDs could be of low density, presenting downstream processing challenges [30].

Hot-melt extrusion (HME) is a process whereby drug, polymer, and additives are heated as the mixture is passed through a rotating screw at controlled temperature, feed rate, and blending speed. The mixed components are melted and extruded to form an amorphous mixture, and the resulting extrudate is milled to form granules. Requirements for the production of amorphous dispersions by HME include components with relatively low melting points and high thermal stability [23,24]. Typically, products resulting from HME are compact granules with suitable flowability and can be robustly produced with little variation between batches [32,33]. Both HME and spray drying are commonly used in the production of commercial products.

While spray drying and HME can handle the majority of compounds, there are cases where neither spray drying nor HME is feasible when the compounds are thermally labile and have low solubility in spray drying solvents [34]. Co-precipitation is a complementary approach that works with compounds that cannot be processed by spray drying and HME [34]. This approach involves the rapid transition of the drug and polymer from one solvent environment in which they are both soluble to another solvent environment in which they are both insoluble. The two streams of solutions are brought in contact under controlled conditions to allow the formation of a drug-polymer co-precipitate in a time scale faster than the API diffusion and crystallization. Co-precipitation of a drug and a polymer has been demonstrated at commercial scales, such as Zelboraf^®^, vemurafenib, from Roche [34].

It is important to understand the impact of the processing route on the physicochemical properties, dissolution, and pharmacokinetic performance of the materials. However, there is a limited number of APIs that can be successfully made into ASD using multiple processing approaches, and of those, only a few have been studied and compared. In the present study, we investigated a model system using posaconazole with hydroxypropylmethylcellulose acetate succinate (medium-fine grade, HPMCAS-MF) amorphous solid dispersions, made by two commonly used approaches, HME and spray drying. Posaconazole, a BCS II weak base drug with low solubility (Table 1) in its crystalline form, benefits from the formulation of the drug as an amorphous solid dispersion to increase its bioavailability. HPMCAS-MF was selected based upon characteristics including physical and chemical stability and performance. Comprehensive characterization of the dispersions and their in vitro performance were evaluated. The ASDs were then filled into capsules and evaluated for their pharmacokinetic performance in cynomolgus monkeys. The aim of this work is to understand how different processing methods impact in vitro and in vivo performance and how in vitro dissolution is correlated to in vivo PK study.

## 2. Materials and Methods

### 2.1. Materials

Posaconazole was manufactured by Merck & Co. Inc. (Kenilworth, NJ, USA). HPLC-grade acetone (Sigma-Aldrich, St. Louis, MO, USA), HPMCAS MF grade (Shin-Etsu Chemical Co., Totowa, NJ, USA) were purchased. All materials were used as received.

### 2.2. Methods

#### 2.2.1. Preparation of SDD Dispersion

The polymer HPMCAS-MF and posaconazole (3:1 wt% of polymer to API ratio) were dissolved in acetone at 5% solid loading. The mixture was stirred on a magnetic stir plate until all components were dissolved. A ProCepT Micro-Spray Dryer with a bifluid nozzle of orifice of 0.6 mm (9060 Zelzate, Belgium) was used in this study. Nitrogen was used as the drying gas at a flow rate of 0.4 m^3^/min. The solution feed rate was 5 mL/min. Inlet and outlet temperatures were set to 95 and 53 °C, respectively. The spray-dried materials were further oven-dried (40 °C under reduced pressure) overnight to remove residual solvent.

#### 2.2.2. Preparation of HME Dispersion

An 18 mm co-rotating twin-screw extruder (Leistritz Corp., Somerville, NJ, USA) in the 40:1 length/diameter (L/D) configuration with a 4.0 mm die at the end of the unit was used for the extrusion. A pre-blended mixture consisting of drug to HPMCAS-MF at a 1:3 wt% ratio was fed into the extruder. A total of 40 g per minute of the blend was fed into the extruder with 200 rpm, and the processing section temperature was controlled at 130 °C. The extrudate was air-cooled and milled via a Fitzmill for downstream evaluation. The samples were stored at 5 °C prior to physical characterization.

#### 2.2.3. Physical Characterization of ASDs

X-ray Powder Diffraction (XRPD). XRPD patterns were collected on a Bruker D8 Advance Powder X-ray Diffractometer (copper X-ray tube, Cu Kα = 1.54 Å, 40 kV, 40 mA, Madison, WI, USA). The powder materials were placed on the standard zero background silicon holders. The XRPD patterns were collected in the angular range of 2–40° 2θ in a step scan mode in Bragg Brentano geometry.

Differential Scanning Calorimetry (DSC). The glass transition temperatures (Tg) of solid dispersions were determined by modulated DSC using TA Instruments (DSC Q2000, New Castle, DE, USA). The solid dispersion powder (~2–5 mg) was loaded on a Tzero pan and sealed with a Tzero hermetic lid with two pinholes on the lid. The heating rate was 2 °C/min with a modulation frequency ± 0.5 °C every 60 s. The Tg was determined by the Universal Analysis software (Version 4.5A) at half height step midpoint.

Scanning Electron Microscopy (SEM). The ASD powder was mounted onto an SEM specimen holder with conductive carbon adhesive tabs (Ted Pella, Inc. Redding, CA, USA) and sputter-coated with 5 nm of platinum in a sputter coater EMS 150T ES (Electron Microscopy Sciences, Hatfield, PA, USA). SEM images were collected using a Hitachi SU5000 SEM (Hitachi High-Technologies Corporation, Tokyo, Japan) with a voltage of 15 kV, spot intensity of 30%, secondary electron (SE) mode, and a working distance of 5.0 mm under high vacuum mode.

X-ray Photoelectron Spectroscopy (XPS). XPS experiments were performed using a Physical Electronics Quantera SXM instrument equipped with a monochromatic Al Kα x-ray source (hν = 1486.6 eV) and a concentric hemispherical analyzer. Charge neutralization was performed using low energy (1.5 eV) electrons and low energy (8 V) Ar^+^ ions. The binding energy axis was calibrated using sputter cleaned Cu foil (Cu 2p3/2 = 932.62 eV) and Au foil (Au 4f7/2 = 83.96 eV). Spectra were charge references to C-(C,H) at 284.8 eV. All measurements were performed at an electron takeoff angle of 45° relative to the sample platen plane.

Samples for Physical Stability Study. Powder of each solid dispersion (~3 g) was added into a 20 mL scintillation vial, and the vial was placed in a 40 °C/75% RH stability chamber under open dish condition for up to 3 months. The sample was subjected to XRPD and DSC tests at each time point (0.5, 1, 2, and 3 months).

#### 2.2.4. In Vitro Dissolution

Simulated gastric fluid (SGF) was made with sodium chloride (34.2 mM), and the pH was adjusted to 1.8 with 12N HCl. The 2× fasted state simulated intestinal fluid (FaSSIF) was prepared using simulated intestinal fluid (SIF) powder (purchased from Biorelevant.com Ltd., London, UK), sodium phosphate monobasic monohydrate (114.5 mM), and sodium chloride (423.7 mM), and the pH was adjusted to 6.9 [35,36].

Two-stage dissolution was performed on the solid dispersion powder at the dose relevant level of drug quantity using the Distek Model 2500 dissolution system (North Brunswick, NJ USA) with USP Apparatus 2 (paddles) setup. The paddles were operated at 100 rpm, and the dissolution medium was maintained at 37 ± 0.5 °C. The SGF medium of 250 mL was added for the first stage, and then FaSSIF medium of 250 mL at 30 min was added for the second stage to achieve the correct pH, ionic strength, and bile salt levels for FaSSIF. The time points that the sample was withdrawn were indicated by the dots in the dissolution plots. At each time point, ~2 mL was sampled and syringe-filtered (0.45 µm PVDF filter). The filtrate was diluted with diluent (water:acetonitrile = 1:1 *v*/*v*) and analyzed by an Agilent HPLC (Santa Clara, CA, USA) with a UV detector. Chromatographic conditions: mobile phase: A = 0.1% phosphoric acid and B = 100% acetonitrile, isocratic 50:50; column length/ID: 20× 4.6 mm; column temperature: 50 °C; detection wavelength: 254 nm; flow rate: 1 mL/min. The HPLC was validated with standard sample with LOD = 0.5 µg/mL and LOQ = 2 µg/mL.

#### 2.2.5. In Vivo Cynomolgus Monkey PK Study

The pharmacokinetic properties of posaconazole amorphous dispersions were assessed in vivo in male cynomolgus monkeys, and studies were conducted under a protocol approved by the Institutional Animal Care and Use Committee (IACUC) of Merck & Co. Inc., Kenilworth, NJ, USA (IACUC Number: 2016-600774-FEB; start date: 18 February 2015). The capsules were prepared by weighing enough amorphous solid dispersion needed for a 40 mg dose to deliver approximately 5 mg/kg. A single-dose administration of the capsule was performed following overnight fasting with free access to water. The capsules were dosed by placing at the end of an 18 Fr gavage tube and pushed out with air. Water (~30 mL) was given following administration. Blood plasma samples were collected before and at 0.25, 0.5, 1, 2, 4, 6, 8, and 24 h after administration into a K-EDTA-coated tube. The samples were diluted with acetonitrile/water (50:50). After centrifugation at 3000 rpm for 5 min, the supernatants were analyzed for posaconazole using HPLC-MS/MS. Chromatographic conditions: mobile phase: A = water and B = acetonitrile, isocratic 50:50; column temperature: ambient; flow rate: 0.4 mL/min. The HPLC-MS/MS was calibrated and validated with standard samples.

## 3. Results and Discussion

### 3.1. Physical Properties of SDD and HME

**Figure 1:**
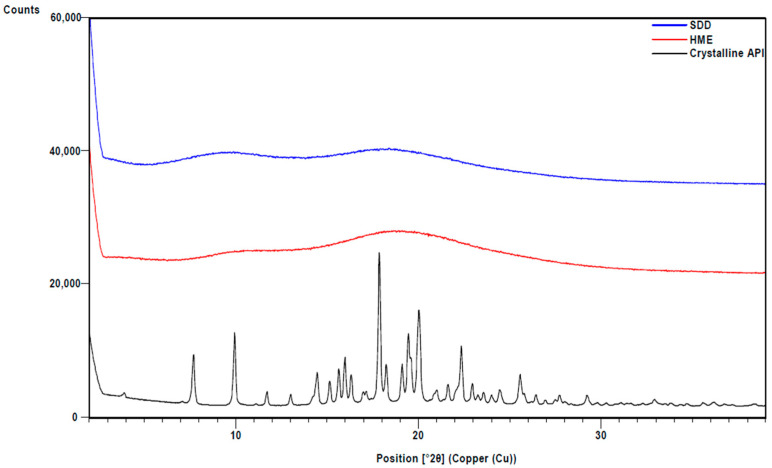
XRPD of HME and SDD solid dispersions. Crystalline API is also shown for comparison.

**Figure 2:**
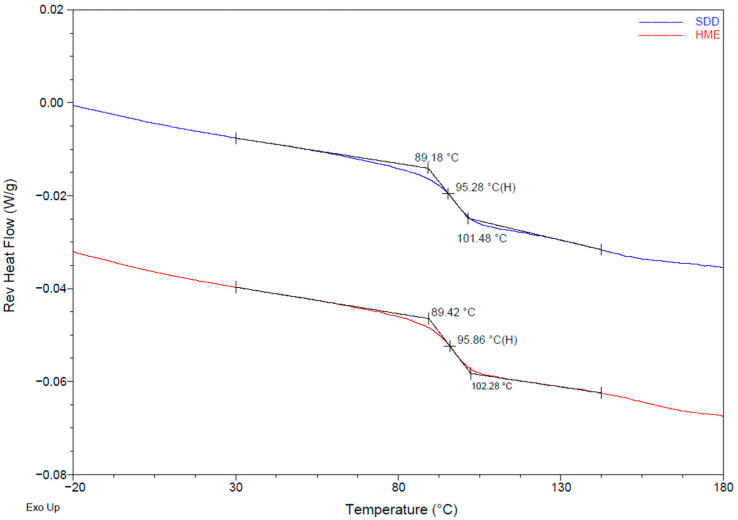
mDSC of HME and SDD solid dispersions shown as reversing heat flow with Tg.

XRPD analysis confirmed that ASDs made via HME and spray drying are amorphous (Figure 1). Modulated DSC was used to measure the glass transition temperature (Tg) of the material and any sign of phase separation. To detect any thermal event, reversing heat flows of the first cycle in modulated DSC are shown in Figure 2. A single Tg was observed in both solid dispersions, indicating that no phase separation occurred. The glass transition temperatures for both ASDs are similar (~96 °C).

**Figure 3:**
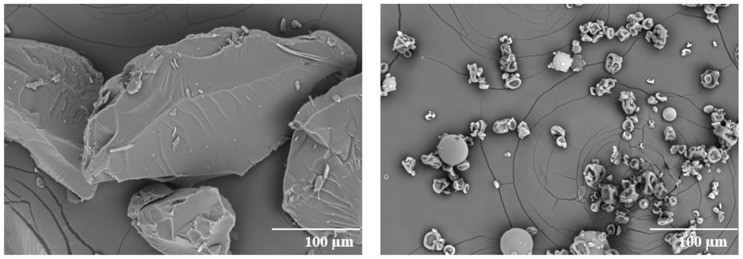
SEM images of ASDs (left: HME; right: SDD) at 300-fold magnification.

The morphology of the ASDs was characterized by SEM (Figure 3). The particles of the HME dispersion show a larger irregular shape with a relatively smooth surface. The SDD particles have raisin-like morphology with smaller particle size. The particle size observed in SEM is consistent with particle size analysis, which gave D50 = 22 µm for SDD and D50 = 157 µm for HME.

To compare the physical stability of the solid dispersions, the materials were subjected to stressing studies at 40 °C/75%RH open dish conditions. The stressed materials were then characterized by XRPD and mDSC. The result (data not included) shows that the dispersions were physically stable for at least three months without any sign of crystallization and phase separation.

### 3.2. Comparison of In Vitro Dissolution of SDD and HME

**Figure 4:**
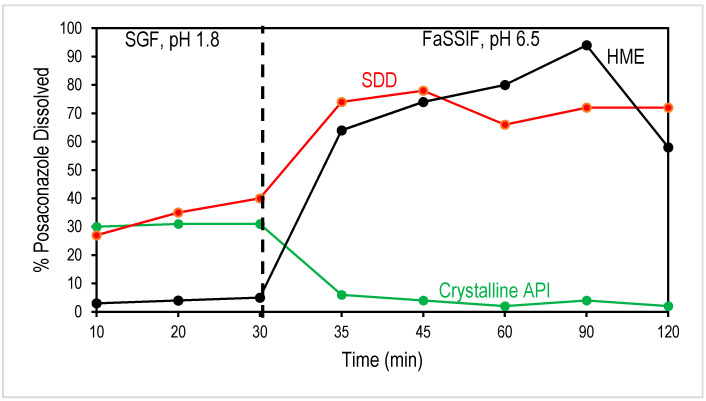
Two-stage biorelevant dissolution of posaconazole ASDs (dissolution of the crystalline API is also shown for comparison). The first 30 min are in SGF (pH 1.8) and then in FaSSIF at a pH of 6.5.

In order to understand the effectiveness of formulating a compound as an amorphous solid dispersion in increasing bioavailability, two-stage biorelevant dissolution can be used to predict the in vivo performance of an oral solid dosage form, using media to mimic the composition and conditions a dosage form would encounter as it moves through the gastrointestinal system. The first stage represents the conditions encountered in the stomach and uses simulated gastric fluid (SGF) with a pH of 1.8. The dosage form is stirred in this solution for 30 min, simulating the amount of time the dosage form would be in the stomach. The API has a relatively high crystalline solubility in this media (~0.79 mg/mL); however, the polymer in the ASD, HPMCAS-MF, has low solubility in acidic media. In amorphous solid dispersions, HPMCAS acts to prevent the posaconazole from dissolving, resulting in a delay in the release of the drug until more basic pH values are reached. In amorphous solid dispersions with high surface areas (smaller particle size) or with high concentrations of API on the surface, the HPMCAS cannot effectively stop the API from dissolving, resulting in the release of the API in the gastric stage [20]. Release of the drug in the stomach has limited benefits, as absorption of the drug into the bloodstream typically occurs after the dosage form is emptied into the intestinal tract. As seen in Figure 4, the HME solid dispersion effectively limits the amount of posaconazole dissolving in the gastric solution. In contrast, SDD shows some release in the SGF, with nearly 40% of the SDD API dissolved after 30 min.

This result is in line with the expected surface areas of HME and SDD, as evidenced by significantly different particle sizes. The SDD particles are much smaller and therefore expected to have significantly higher surface areas. The higher surface area can result in more API being exposed at the surface of the amorphous dispersion, and therefore available to be dissolved, even if the polymer is insoluble. A similar observation of in vitro dissolution and particle size effect of SDD and HME has been reported [20].

In addition to the surface area, the surface composition with respect to the bulk can also play a role in API release in the SGF. XPS was run on the various solid dispersions to compare the surface composition of the powders to the bulk composition (Table 2). The ASDs have a 25% posaconazole bulk drug loading; XPS analysis of both SDD and HME indicates that the SDD and HME have comparable, albeit slightly low, posaconazole compositions compared to the bulk at 18% and 21%, respectively. Therefore, the increased amount of posaconazole released in the SGF stage for the SDD particles is related primarily to the higher surface area compared to the HME. For reference, crystalline API is compared to the ASDs. As expected, the percent dissolved is high for the crystalline API in the SGF media, in line with the higher solubility in acidic media. However, upon the introduction of FaSSIF, the percent dissolved significantly drops to approximately the crystalline solubility.

Interestingly, in the FaSSIF stage, both SDD and HME show comparable drug release, indicating that API release in the SGF stage does not significantly impact drug concentration in the FaSSIF stage. This can be explained by the effectiveness of HPMCAS in maintaining drug supersaturation. When transitioned to the FaSSIF stage, the dissolved HPMCAS acts as a crystallization inhibitor that prevents dissolved drugs in the SGF stage from crystallizing out, as in the case of SDD. In addition, the drug concentration toward the end of the FaSSIF stage, particularly for HME, indicated possible API crystallization. Depending on the absorption rate of the API, the decrease at the ~2 h time point may not be biorelevant as the level of supersaturation would be reduced as the drug is absorbed. As a BCS class II compound, posaconazole absorption may be fast enough that precipitation in vivo is minimized. Therefore, complimentary pharmacokinetic data will provide additional insight beyond the in vitro dissolution data.

### 3.3. Comparison of In Vivo Performance of SDD and HME

**Figure 5:**
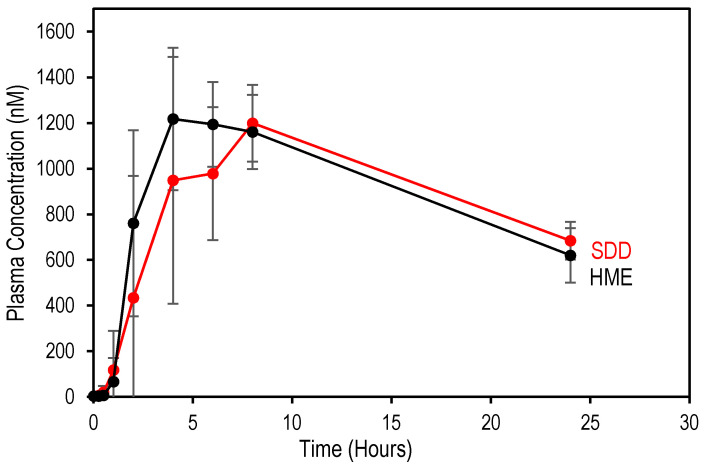
Mean posaconazole blood plasma concentrations following administration of SDD and HME formulations at 5 mg/kg body weight in cynomolgus monkey (*n* = 6).

Posaconazole ASDs were filled into capsules and tested for their in vivo performance in cynomolgus monkeys. The pharmacokinetic data are reported in Table 3, and mean posaconazole plasma concentration profiles are shown in Figure 5, respectively. Notably, the AUC and Cmax values for SDD and HME are similar. These data are aligned with the observed dissolution data. In particular, the SDD and HME show similarly high percent dissolved in dissolution for the second stage (FaSSIF). As expected, the API release in the first stage for the SDD material does not impact the maximum concentration of the API in the cynomolgus monkeys. This further confirms that dissolved HPMCAS in the FaSSIF stage of in vitro dissolution successfully prevents dissolved API in the SGF stage from precipitating out.

Importantly, this data supports the conclusion that the processing route impacts the in vitro performance of posaconazole amorphous solid dispersions. However, in vivo, the SDD and HME perform similarly in cynomolgus monkeys and overall agree with the FaSSIF stage dissolution data.

## 4. Conclusions

Two processing methods were used to develop amorphous solid dispersions of posaconazole: HME and spray drying. The impact of processing methods on in vitro and in vivo performance was compared. In vitro two-stage dissolution shows that significant drug release was observed in the SGF stage with SDD but not with HME. Smaller particle size (larger surface area) may contribute to drug release in the SGF stage for SDD. In the FaSSIF stage, HME and SDD have similar drug concentrations, indicating that released drugs in the SGF stage for SDD were not crystallized out when transitioned to the FaSSIF stage due to dissolved HPMCAS, which inhibits drug crystallization. The in vivo cynomolgus monkey study shows that HME and SDD performed similarly based on AUC and Cmax, indicating drug release in the SGF stage does not impact in vivo performance, which is consistent with the in vitro results in the FaSSIF stage. The paper highlights the importance of evaluating processing approaches for amorphous solid dispersions to compare their in vitro and in vivo performance.

## Figures and Tables

**Table 1:** Physical/chemical properties of posaconazole.

**Chemical Structure**	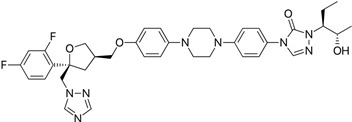
**Melting Point (°C)**	167.9
**pKa**	3.6 (piperazine N) and 4.6 (triazole N)
**Log P**	5
**BCS Classification**	II
**Solubility (mg/mL)**	pH 1: 0.79 pH 3: 0.003 pH 7: 0.001 Fasted state simulated intestinal fluid (FaSSIF): 0.002

**Table 2:** XPS data for SDD and HME that have 25% drug loading with HPMCAS based on the atomic fraction of nitrogen (N) and fluorine (F), which are absent in the polymer HPMCAS.

Sample	~API Loading on Surface Based on F and N
SDD	18%
HME	21%
Posaconazole standard	100%
HPMCAS standard	0%

**Table 3:** Pharmacokinetic data for posaconazole amorphous solid dispersions in cynomolgus monkeys.

Sample	C_max_ (nmol/L)	AUC (h·nmol/L)	T_max_ (h)	C_max_ Ratio *	AUC Ratio *
SDD	1267 ± 217	18,147 ± 5889	6	0.98	0.85
HME	1293 ± 100	21,423 ± 1285	4	1.00	1.00

* C_max_ ratio and AUC ratio were calculated based on HME C_max_ and AUC.
